# Supplementary material for: Cost-effectiveness of vector control for supplementing mass drug administration for eliminating lymphatic filariasis in India
Source: PLoS Negl Trop Dis. 2024 Dec 4;18(12):e0011835. doi: 10.1371/journal.pntd.0011835 (PMC11649082; doi:10.1371/journal.pntd.0011835)
Supplement: S1 Text — Fig. A: Discussion of study plans at the Field Unit. Fig. B: Researchers visit a study village to review lymphatic filariasis control. Table A: Microfilaria prevalence (MfP) by village and year. Table B: Antigen prevalence (AgP) by village and year. Table C: Average prevalence of MfP and AgP in base year (2010) and final year (2013) by condition. Text A: Sensitivity analysis. Table D: Incremental costs and change in MfP prevalence. Table E: Incremental percentage reduction in MfP by condition compared to MDA. Table F: Sensitivity analysis of incremental cost-effectiveness ratios of vector control conditions (dollars per person per year per percentage point change in MfP prevalence). Table G: Prevalence of lymphatic filariasis globally. In India, and in Tamil Nadu, 1990–2021. Fig. C: Distributions of base and final years’ LF prevalence by condition. (DOCX) [file pntd.0011835.s001.docx]

**S1 Text (Supporting Information)**

**Cost-effectiveness of vector control for supplementing mass drug administration for eliminating lymphatic filariasis in India**

Short title: Cost-effectiveness of strategies for lymphatic filariasis elimination in India

Donald S. Shepard Ph.D.^1^

Aung K. Lwin M.D., M.S.^1^

Sunish I. Pulikkottil Ph.D. ^2,3^

Mariapillai Kalimuthu M.Sc.. M.Phil.^2^

Natarajan Arunachalam Ph.D.^2^

Brij K. Tyagi Ph.D.^2,4^

Graham B. White Ph.D.^5^

^1^ Heller School for Social Policy and Management, MS035, Brandeis University, Waltham, Massachusetts 02454-9110, USA

^2^ Indian Council of Medical Research (ICMR) Centre for Research in Medical Entomology, Field Station, 4-Sarojini Street, Chinna Chokkikulam, Madurai 625 002, Tamil Nadu, India

^3^ Regional Medical Research Centre (ICMR), Port Blair 744103, Andaman & Nicobar Islands, India

^4^ Department of Zoology & Environment Science, Punjabi University,

Patiala, Punjab 147002, India

^5^ Department of Entomology and Nematology, University of Florida, P.O. Box 110620, Gainesville, Florida 32611-0620, USA.

Corresponding author: Donald S. Shepard, Heller School for Social Policy and Management, MS035, Brandeis University, Waltham, Massachusetts 02454-9110, USA. Tel: +1-617-584-6664, E-mail: shepard@brandeis.edu

**S1 Text (Supporting Information)**

**Contents**

Fig. A in S1 Text: Discussion of study plans at the Field Unit

Fig. B in S1 Text: Researchers visit a study village to review lymphatic filariasis control

Table A in S1 Text: Microfilaria prevalence (MfP) by village and year

Table B in S1 Text: Antigen prevalence (AgP) by village and year

Table C in S1 Text: Average prevalence of MfP and AgP in base year (2010) and final year (2013) by condition

Text A in S1 Text: Sensitivity analysis

Table D in S1 Text. Incremental costs and change in MfP prevalence

Table E in S1 Text: Incremental percentage reduction in MfP by condition compared to MDA

Table F in S1 Text: Sensitivity analysis of incremental cost-effectiveness ratios of vector control conditions (dollars per person per year per percentage point change in MfP prevalence)

Table G in S1 Text: Prevalence of lymphatic filariasis globally. in India, and in Tamil Nadu, 1990-2021

Fig. C in S1 Text: Distributions of base and final years’ LF prevalence by condition


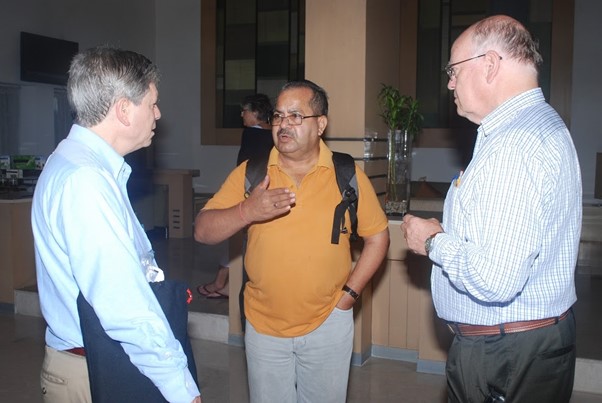
**Fig. A in S1 Text:** **Discussion of study plans at the Field Unit.** From left to right: Prof. Donald S. Shepard (Brandeis University, USA), Dr B .K. Tyagi (Indian Council of Medical Research, Centre for Research on Medical Entomology, India) and Prof. Graham B. White (University of Florida, USA).


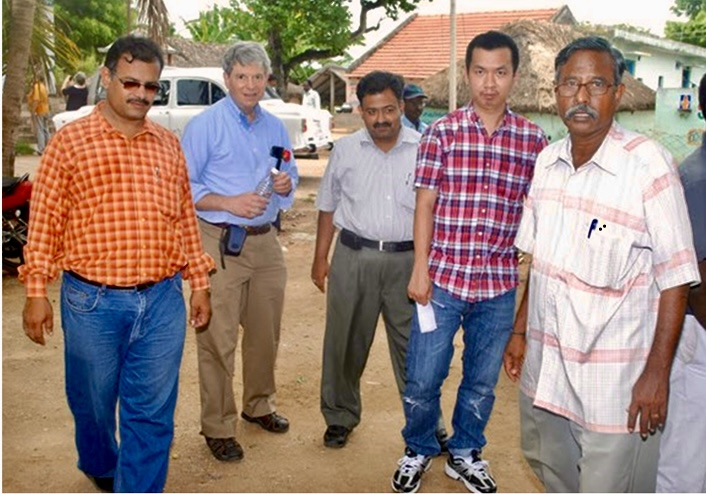
**Fig. B in S1 Text:** **Researchers visit a study village to review lymphatic filariasis control.** From left to right: Shri Ranganathan Krishnamoorthi of Indian Council on Medical Research, Centre for Research on Medical Entomology (ICMR-CRME), India, Field Unit, Prof. Donald S. Shepard (Brandeis University, USA), Dr. Sunish Pulikkottil (ICMR-CRME Field Unit), Dr. Aung K. Lwin (Brandeis University, USA), and Shri A. Munirathinam (ICMR-CRME Field Unit). Photographer: Dr. B. K. Tyagi (Director, ICMR-CRME).

| **Table A in S1 Text: Microfilaria prevalence (MfP) by village and year** | | | | | | |
| --- | --- | --- | --- | --- | --- | --- |
|  | **Village** | **Condition** | **2010** | **2011** | **2012** | **2013** |
| 1 | Aruthangudi | MDA | 2.551 | 0.529 | 0.529 | 0.562 |
| 2 | Chozhapandipuram | MDA | 2.709 | 1.720 | 1.525 | 0.700 |
| 3 | Edaiyur | MDA | 0.633 | 0.330 | 0.000 | 0.000 |
| 4 | Elrampattu | MDA | 4.590 | 4.502 | 2.181 | 0.388 |
| 5 | Eravalam | MDA | 0.813 | 0.000 | 0.699 | 0.000 |
| 6 | Koovanur | MDA | 3.974 | 0.971 | 0.971 | 0.000 |
| 7 | Meiyur | MDA | 5.952 | 1.899 | 1.818 | 0.676 |
| 8 | Melathazhanur | MDA | 1.587 | 2.890 | 1.124 | 1.220 |
| 9 | Memalur | MDA | 1.872 | 1.458 | 0.000 | 0.000 |
| 10 | Sivanarthangal | MDA | 10.769 | 5.917 | 5.000 | 4.301 |
| 11 | Thimmechur | MDA | 4.124 | 1.894 | 2.591 | 0.000 |
| 12 | Veeratagaram | MDA | 6.628 | 2.527 | 2.848 | 0.826 |
| 13 | Alur | VCS | 3.241 | 0.935 | 0.552 | 0.000 |
| 14 | Athandamaruthur | VCS | 1.382 | 0.000 | 0.000 | 0.000 |
| 15 | Avi pudhur | VCS | 5.455 | 3.175 | 1.587 | 0.000 |
| 16 | Aviyur | VCS | 3.008 | 0.699 | 0.000 | 0.000 |
| 17 | Kacchikuppam | VCS | 5.983 | 3.846 | 2.759 | 5.217 |
| 18 | Kottamedu | VCS | 3.960 | 3.333 | 0.000 | 0.000 |
| 19 | Millaripattu | VCS | 0.862 | 0.000 | 0.000 | 0.000 |
| 20 | Nedumudaiyan | VCS | 3.431 | 1.667 | 1.869 | 0.939 |
| 21 | Padiyeandal | VCS | 5.833 | 2.203 | 1.128 | 2.212 |
| 22 | Senganankollai | VCS | 1.322 | 0.889 | 0.000 | 0.000 |
| 23 | Thagadi | VCS | 4.502 | 3.415 | 2.535 | 1.700 |
| 24 | Thanaganandal | VCS | 5.755 | 2.740 | 4.242 | 1.550 |
| 25 | Avi kolapakkam | VCI | 1.810 | 0.498 | 0.481 | 0.000 |
| 26 | Kattupaiyur | VCI | 6.707 | 3.188 | 1.374 | 1.370 |
| 27 | Keezhathazhanur | VCI | 1.282 | 0.000 | 0.680 | 0.000 |
| 28 | Kolaparai | VCI | 1.911 | 0.000 | 0.565 | 0.000 |
| 29 | Mohalar | VCI | 0.424 | 0.000 | 0.344 | 0.000 |
| 30 | Nariyeantal | VCI | 5.825 | 7.692 | 4.464 | 0.000 |
| 31 | Pazhangur | VCI | 2.685 | 0.977 | 0.521 | 0.372 |
| 32 | Pudhur | VCI | 3.226 | 0.000 | 1.429 | 0.000 |
| 33 | Rajampalayam | VCI | 2.151 | 0.000 | 0.000 | 0.000 |
| 34 | T.Keeranur | VCI | 1.190 | 0.000 | 0.000 | 0.000 |
| 35 | Thurinjupattu | VCI | 4.545 | 0.909 | 0.000 | 0.446 |
| 36 | Tirupalapandal | VCI | 0.730 | 0.379 | 0.000 | 0.400 |

| **Table B in S1 Text: Antigen prevalence (AgP) by village and year** | | | | | | |
| --- | --- | --- | --- | --- | --- | --- |
|  | **Village** | **Condition** | **2010** | **2011** | **2012** | **2013** |
| 1 | Aruthangudi | MDA | 5.550 | 0.000 | 10.260 | 0.000 |
| 2 | Chozhapandipuram | MDA | 9.520 | 8.240 | 10.230 | NA |
| 3 | Edaiyur | MDA | 15.520 | 12.730 | 5.260 | 0.000 |
| 4 | Elrampattu | MDA | 8.620 | 5.450 | 18.870 | 5.172 |
| 5 | Eravalam | MDA | 4.170 | 0.000 | 4.000 | 0.000 |
| 6 | Koovanur | MDA | 13.330 | 12.730 | 6.780 | 5.000 |
| 7 | Meiyur | MDA | 10.000 | 11.110 | 10.000 | 0.000 |
| 8 | Melathazhanur | MDA | 3.125 | 13.790 | 21.880 | 6.250 |
| 9 | Memalur | MDA | 1.560 | 6.350 | 11.590 | NA |
| 10 | Sivanarthangal | MDA | 11.760 | 13.790 | 0.000 | 5.556 |
| 11 | Thimmechur | MDA | 8.330 | 8.890 | 2.500 | 0.000 |
| 12 | Veeratagaram | MDA | 5.450 | 11.760 | 15.380 | 1.818 |
| 13 | Alur | VCS | 7.890 | 8.570 | 8.570 | 0.000 |
| 14 | Athandamaruthur | VCS | 15.790 | 11.430 | 13.890 | 0.000 |
| 15 | Avi pudhur | VCS | 16.670 | 0.000 | 0.000 | 0.000 |
| 16 | Aviyur | VCS | 3.700 | 0.000 | 0.000 | 0.000 |
| 17 | Kacchikuppam | VCS | 27.270 | 10.530 | 16.670 | 4.550 |
| 18 | Kottamedu | VCS | 16.670 | 5.560 | 0.000 | 0.000 |
| 19 | Millaripattu | VCS | 10.000 | 11.110 | 5.260 | 0.000 |
| 20 | Nedumudaiyan | VCS | 5.560 | 16.130 | 9.090 | 2.630 |
| 21 | Padiyeandal | VCS | 4.760 | 9.760 | 10.200 | 0.000 |
| 22 | Senganankollai | VCS | 14.630 | 7.690 | 2.380 | 0.000 |
| 23 | Thagadi | VCS | 20.000 | 7.690 | 7.690 | NA |
| 24 | Thanaganandal | VCS | 4.170 | 17.390 | 21.430 | 11.538 |
| 25 | Avi kolapakkam | VCI | 5.410 | 5.710 | 5.000 | 7.890 |
| 26 | Kattupaiyur | VCI | 8.200 | 6.560 | 10.770 | 0.000 |
| 27 | Keezhathazhanur | VCI | 19.230 | 4.350 | 3.850 | 3.570 |
| 28 | Kolaparai | VCI | 17.240 | 3.700 | 0.000 | 0.000 |
| 29 | Mohalar | VCI | 2.130 | 11.360 | 7.270 | 2.170 |
| 30 | Nariyeantal | VCI | 16.670 | 6.670 | 9.090 | 0.000 |
| 31 | Pazhangur | VCI | 10.710 | 15.090 | 2.900 | 0.000 |
| 32 | Pudhur | VCI | 25.000 | 0.000 | 0.000 | 0.000 |
| 33 | Rajampalayam | VCI | 0.000 | 0.000 | 20.000 | 5.560 |
| 34 | T.Keeranur | VCI | 11.110 | 0.000 | 0.000 | 5.560 |
| 35 | Thurinjupattu | VCI | 15.000 | 2.560 | 3.850 | 2.380 |
| 36 | Tirupalapandal | VCI | 0.000 | 2.080 | 3.570 | 0.000 |

Note: NA denotes not available because AgP was not collected in that year.

**Table C in S1 Text: Average prevalence of MfP and AgP in base year (2010) and final year (2013) by condition**

| **Condition** | **MfP** | **MfP** | **AgP** | **AgP** |
| --- | --- | --- | --- | --- |
|  | **2010** | **2013** | **2010** | **2013** |
| MDA | 3.9 | 0.7 | 8.1 | 2.4 |
| VCS | 3.7 | 1.0 | 12.3 | 1.7 |
| VCI | 2.7 | 0.2 | 10.9 | 2.3 |

**Text A** **in S1 Text:** **Sensitivity analysis**

Table D in S1 Text illustrates the additional costs associated with implementing vector control interventions (VCS and VCI) compared to the standard MDA approach, along with the corresponding decrease in MfP prevalence from 2010 to 2013. While VCI demonstrated a greater reduction in prevalence, both MDA and VCS also showed substantial gains at their best estimates (upper bound of the reduction). Confidence intervals were calculated to account for the potential variability in these gains.

**Table D in S1 Text: Incremental costs and change in MfP prevalence**

| Condition | Annual cost per person ($) | Incremental annual cost per person ($) | % reduction in MfP from 2010 to 2013 | Lower bound | Upper bound |
| --- | --- | --- | --- | --- | --- |
| MDA | 0.53 | 0.00 | 82.9 | 67.7 | 98.1 |
| VCS | 1.02 | 0.49 | 83.7 | 67.4 | 100.0 |
| VCI | 1.83 | 1.30 | 95.6 | 90.9 | 100.0 |

Table E in S1 Text presents the incremental percentage change in prevalence from 2010 to 2013. For each village with MfP prevalence in 2010 and 2013, the percentage reduction was calculated. As three villages with EPB did not have values for 2013, they were excluded from this calculation. Then the percentage changes were averaged by arm to give the average column in S6b Table. Note that each arm had 9 – 12 villages with data at both points. To generate upper and lower bounds, a normal distribution was applied to these percentage changes and 95% confidence intervals (1.96 standard errors in either direction from the mean. These results highlighting the relative effectiveness of each intervention compared to MDA. The integrated vector control method (VCI) exhibited a substantially larger reduction in prevalence, whereas VCS achieved only a modest decline.

**Table E in S1 Text: Incremental percentage reduction in MfP by condition compared to MDA**

| Condition | Incremental reduction in MfP | Upper bound | Lower bound |
| --- | --- | --- | --- |
| MDA | 0.0 | 0.0 | 0.0 |
| VCS | 0.8 | 32.3 | -30.7 |
| VCI | 12.7 | 32.6 | -7.2 |

Table F in S1 Text displays the results of a sensitivity analysis, assessing the potential fluctuations in the effectiveness of vector control interventions in reducing MfP prevalence. The central value uses the mean cost in that condition divided by the mean change in MfP among villages in that condition with observations at both time periods The most and least favorable columns are based on 95% confidence intervals on changes in MFP with each arm assuming normal distributions. The most favorable cost-effectiveness ratio assumes a reduction equal to the largest possible reduction in MfP, based on the upper confidence interval for that arm, paired with the smallest possible reduction for the MDA arm. Similarly, the least favorable cost-effectiveness ratio assumes a reduction equal to the smallest possible reduction in MfP, based on the lower confidence interval for that arm paired with the largest possible reduction for the MDA arm. With only 12 villages with data for MDA and VCS and only 9 for VCI, the confidence intervals are necessarily wide, so none of the differences were statistically significant.

**Table F in S1 Text: Sensitivity analysis of incremental cost-effectiveness ratios of vector control conditions (dollars per person per year per percentage point change in MfP prevalence)**

| Vector control condition | Incremental cost-effectiveness | Most favorable | Least favorable |
| --- | --- | --- | --- |
| VCS | 0.62 | 0.02 | Not cost effective |
| VCI | 0.10 | 0.04 | Not cost effective |
|  |  |  |  |

**Table G in S1 Text:** **Prevalence of lymphatic filariasis globally, in India, and in Tamil Nadu, 1990-2021***

|  | **Global** | | | I**ndia** | | | **Tamil Nadu** | | |
| --- | --- | --- | --- | --- | --- | --- | --- | --- | --- |
| **Year** | **Upper** | **Value** | **Lower** | **Upper** | **Value** | **Lower** | **Upper** | **Value** | **Lower** |
| 1990 | 4.7% | 3.9% | 3.4% | 12.3% | 11.4% | 10.6% | 4.3% | 3.5% | 2.9% |
| 1991 | 4.5% | 3.9% | 3.4% | 12.0% | 11.1% | 10.3% | 4.3% | 3.5% | 2.9% |
| 1992 | 4.4% | 3.9% | 3.4% | 11.7% | 10.8% | 10.0% | 4.3% | 3.5% | 2.9% |
| 1993 | 4.4% | 3.9% | 3.4% | 11.4% | 10.5% | 9.7% | 4.3% | 3.5% | 2.8% |
| 1994 | 4.4% | 3.9% | 3.4% | 11.1% | 10.3% | 9.5% | 4.2% | 3.4% | 2.8% |
| 1995 | 4.5% | 3.9% | 3.4% | 10.9% | 10.1% | 9.3% | 4.2% | 3.4% | 2.8% |
| 1996 | 4.4% | 3.9% | 3.4% | 10.7% | 9.9% | 9.1% | 4.2% | 3.3% | 2.8% |
| 1997 | 4.3% | 3.8% | 3.5% | 10.5% | 9.7% | 8.9% | 4.1% | 3.3% | 2.7% |
| 1998 | 4.3% | 3.8% | 3.4% | 10.3% | 9.4% | 8.7% | 4.0% | 3.2% | 2.7% |
| 1999 | 4.3% | 3.8% | 3.4% | 10.0% | 9.2% | 8.5% | 3.9% | 3.2% | 2.6% |
| 2000 | 4.2% | 3.7% | 3.3% | 9.8% | 9.0% | 8.3% | 3.8% | 3.1% | 2.5% |
| 2001 | 4.1% | 3.6% | 3.3% | 9.6% | 8.8% | 8.0% | 3.7% | 3.0% | 2.4% |
| 2002 | 3.7% | 3.4% | 3.1% | 9.2% | 8.4% | 7.7% | 3.5% | 2.8% | 2.3% |
| 2003 | 3.4% | 3.1% | 2.9% | 8.8% | 8.1% | 7.4% | 3.2% | 2.5% | 2.1% |
| 2004 | 3.1% | 2.8% | 2.6% | 8.4% | 7.7% | 7.0% | 2.9% | 2.3% | 1.8% |
| 2005 | 3.0% | 2.6% | 2.4% | 8.0% | 7.3% | 6.6% | 2.6% | 2.0% | 1.6% |
| 2006 | 2.7% | 2.4% | 2.2% | 7.4% | 6.7% | 6.1% | 2.2% | 1.7% | 1.4% |
| 2007 | 2.3% | 2.1% | 1.9% | 6.5% | 6.0% | 5.4% | 1.8% | 1.4% | 1.1% |
| 2008 | 2.0% | 1.8% | 1.6% | 5.7% | 5.1% | 4.6% | 1.4% | 1.1% | 0.9% |
| 2009 | 1.7% | 1.5% | 1.4% | 5.0% | 4.5% | 4.0% | 1.1% | 0.8% | 0.7% |
| 2010 | 1.6% | 1.4% | 1.2% | 4.6% | 4.1% | 3.6% | 1.0% | 0.7% | 0.6% |
| 2011 | 1.5% | 1.3% | 1.2% | 4.6% | 4.1% | 3.6% | 0.9% | 0.7% | 0.6% |
| 2012 | 1.4% | 1.3% | 1.2% | 4.8% | 4.3% | 3.8% | 0.9% | 0.7% | 0.6% |
| 2013 | 1.4% | 1.3% | 1.2% | 5.1% | 4.5% | 4.0% | 0.9% | 0.7% | 0.6% |
| 2014 | 1.5% | 1.3% | 1.2% | 5.3% | 4.8% | 4.3% | 0.9% | 0.7% | 0.6% |
| 2015 | 1.5% | 1.3% | 1.2% | 5.4% | 4.8% | 4.3% | 0.9% | 0.7% | 0.6% |
| 2016 | 1.4% | 1.2% | 1.1% | 5.1% | 4.5% | 4.0% | 0.9% | 0.7% | 0.6% |
| 2017 | 1.2% | 1.0% | 0.9% | 4.3% | 3.8% | 3.4% | 0.9% | 0.7% | 0.5% |
| 2018 | 1.0% | 0.9% | 0.8% | 3.5% | 3.0% | 2.7% | 0.9% | 0.7% | 0.5% |
| 2019 | 0.9% | 0.8% | 0.7% | 3.0% | 2.6% | 2.3% | 0.9% | 0.7% | 0.5% |
| 2020 | 0.9% | 0.8% | 0.6% | 2.9% | 2.5% | 2.2% | 0.8% | 0.6% | 0.5% |
| 2021 | 0.9% | 0.7% | 0.6% | 2.7% | 2.4% | 2.1% | 0.8% | 0.6% | 0.5% |

* Source: Derived from Global Burden of Diseases study [58] (see main text for citation). This table was used to generate Figure 6 in main text. Note: Upper and lower denote upper and lower certainty bounds, respectively. Value denotes best estimate of prevalence.


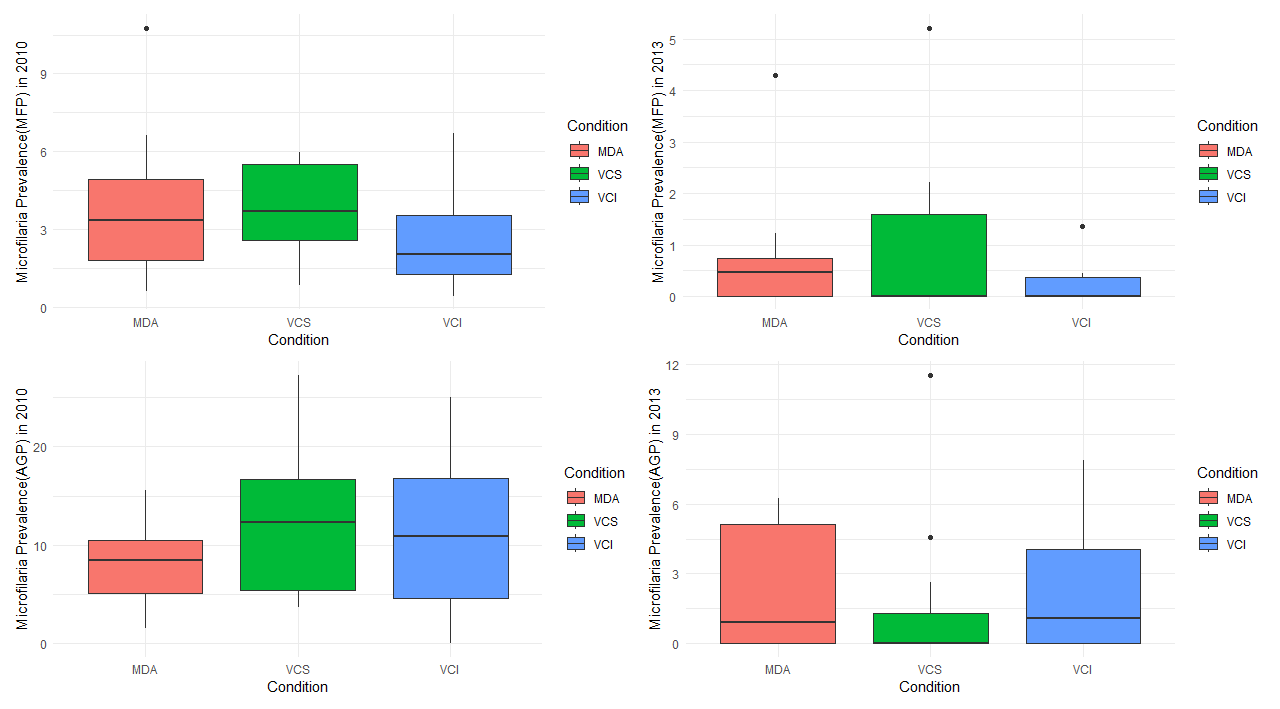
**Fig. C in S1 Text: Distributions of base and final years’ LF prevalence by condition**
