# Supplementary material for: Cost-effectiveness of vector control for supplementing mass drug administration for eliminating lymphatic filariasis in India
Source: PLoS Negl Trop Dis. 2024 Dec 4;18(12):e0011835. doi: 10.1371/journal.pntd.0011835 (PMC11649082; doi:10.1371/journal.pntd.0011835)
Supplement: Attachment — Submitted filename: Comments on cost paper_KK.docx [file pntd.0011835.s002.docx]

Title: Cost-effectiveness of vector control strategies for supplementing mass drug administration for eliminating lymphatic filariasis in India

General comment:

The present manuscript deals with the results of a study, conducted as a follow-up of cost-effectiveness of Mass Drug Administration (MDA) with or without vector control (Krishnamoorthy et al., 2002) in 36 different villages in Kallakurichi health district between 2010 and 2013. While MDA was administered by the State Health department as per the national guidelines, vector control was carried out by the research team with community participation. Though the findings were similar to the earlier publication of cost-effectiveness of MDA and MDA+VC, the current publication has additionally used filarial antigenemia and estimated DALYs averted. MDA cost analysis tool (MDA-CAT) and VC cost analysis tool (VC-CAT) were employed, to enter the cost data and perform cost analyses. Two interventions against the vector, one with expanded polystyrene beads (EPBs) in breeding places and the other integrated this with pyrethroid impregnated curtains (PIC) were carried out to compare the outcome of MDA with vector control strategies. The study also confirmed that MDA is more cost-effective in achieving interruption of transmission. The manuscript with added outcome indicators is recommended for publication. Though the manuscript is published after a gap of 10 years it provides valuable information on the impact of vector control interventions4.

Specific comments:

1. The present document added another arm with integrated vector control. Vector control included only treatment of cesspits with polystyrene beads (VCS) and integrated with use of insecticide treated curtains VCI). Earlier trial used application of *Bacillus sphaericus* in breeding places. It is understood that this intervention was not used in the present study.
2. Line 58 – add after additional benefit “on infection prevalence”
3. Line 60 – refer to the latest reference on WHO-targeted NTDs for control.
4. Line 61-63 – Indian states started MDA programme in 2004 and earlier studies were trails with MDA in selected districts. This can be modified.
5. Replace the reference 7 with the latest one “Vol. 98, No. 41, pp. 489 – 502” - 19 countries and territories (Cambodia, the Cook Islands, Egypt, Kiribati, Maldives, Malawi, Marshall Islands, Niue, Palau, Sri Lanka, Thailand, Togo, Tonga, Vanuatu, Viet Nam, Wallis and Futuna, Yemen, Lao PDR and Bangladesh) are now acknowledged as achieving elimination of lymphatic filariasis as a public health problem.
6. Line 79 – replace “elimination from 20 countries” with “19 countries validated for elimination of LF as a public health problem (Vol. 98, No. 41, pp. 489 – 502 and reference 8)
7. Line 81 – add “and elimination of LF in 2004” after 1955.
8. Line 81 – replace “Control” by elimination
9. Line 86-89 – Rephrase the sentences as “Initially the MDA program administered a single dose of diethylcarbamazine (DEC) to each eligible person and subsequently administered DEC plus albendazole annually to the whole population of districts (implementation units) where LF was endemic [10, 11]”.
10. Line 118 - Add a suitable reference after COVID-19
11. Line 128 – add “present” after “study”
12. Line 220 – rephrase as “anti-filarial drugs with DEC and albendazole…”
13. Line 230 – add “unused” before wells
14. Line 238 – what modification was done in unused wells?
15. Line 315 – the method of calculating DALY per 1000 population averted can be given
16. Line 352 – add “infection” after LF
17. Line 355 – justify use of LF prevalence data for calculating DALYs per person averted
18. Figure 5 – correct the y axis title as AgP
19. Line 416 – 418 & Figure 4 and 5 - what is the explanation for significant reduction in AgP following the intervention?
20. Line 428 – the benefit of reduction in vector density following VCS and VCI can be mentioned
21. Line 432 - provide the source of data for prevalence of infection in India
22. Line 434 and Figure 6 – the limitation of using prevalence data from sample survey in selected sites every year can be explained, particularly for India
23. Line 436 – how to justify estimating people affected by LF based on prevalence which is expected to be done only from endemic areas
24. Table 7 – how do we explain the change in AgP from 2010 (baseline) and 2013 (post intervention)
25. Line 445 – justify the extrapolation of data from 36 villages to the whole of nation
26. Line 455 – add “infection” after LF
27. Line 458 – replace the word “affected” by infected
28. Line 461 – add “Kalakurichi district” after the study
29. Line 468 – add “selected” before endemic (MDA was conducted in selected districts in 1996-98 and LF elimination programme was introduced in India by 2004)
30. Line 469 – replace 2001 by 2007 as nation-wide co-administration of DEC+albendazole was introduced in India by 2007. (trial with DEC+albendazole, as co-administration was done in 2001 in selected districts in Tamil Nadu)
31. Line 472 – add “Kalakurich health unit district of” before Villupuram
32. Line 476 – rephrase the sentence as “reached the target levels of AgP<2% in children by transmission assessment survey”
33. Line 491 – add replace “the only” with one of the comparative studies
34. Line 493 – replace “progress” by “impact”
35. Line 495 – add “short term” before study
36. Line 506 – add “reduced” before MfP and replace adequately controlled with “in selected sites”
37. Line 507 – replace “most of the” with “at least 99” districts by giving reference “Roy N. Elimination of lymphatic filariasis – India: Updates and way forward. MJMS. 2018; 3(2): 1-3”.
38. Line 508 – replace the sentence “However, 12 such villages in non-endemic areas had MfP between 3.2% and 11.2% with “Remapping of non-endemic areas in India [60] suggest prevalence of infection above 1% in 12 such sites” (consider deleting this paragraph line 505-509 which is not relevant here)
39. Line 511 – provide data on reduction in mosquito nuisance
40. Line 535 – add “in achieving the interruption of transmission” after MDA
