# Supplementary material for: Cost-effectiveness of vector control for supplementing mass drug administration for eliminating lymphatic filariasis in India
Source: PLoS Negl Trop Dis. 2024 Dec 4;18(12):e0011835. doi: 10.1371/journal.pntd.0011835 (PMC11649082; doi:10.1371/journal.pntd.0011835)
Supplement: Attachment — Submitted filename: Reviewer comments_Response_7.docx [file pntd.0011835.s004.docx]

***Response to reviewers and editor regarding: “Cost-effectiveness of vector control strategies for supplementing* mass drug administration for eliminating lymphatic filariasis in India”**

Authors’ note: We thank the editor and reviewers for the thoughtful comments and granting the additional time needed for our response. For clarity we have applied decimal numbering to the reviewers’ comments and begin our response to each comment with the label “Authors:”. As line and reference numbers may change during editing, the numbers in this response are approximate. Our revision also incorporates additional edits , clarifications and the requested original data (Supporting Information S3 Table and S4 Table). In addition, all necessary release and permission forms from the subjects, photographers, and the creator of the map (Figure 1) have been uploaded to the journal’s portal.

**Reviewer #1:**

1.1	Major comments: The paper presents a cost-effectiveness analysis of vector control strategies for supplementing mass drug administration for eliminating lymphatic filariasis in India. Overall, the cost data were clearly presented and comprehensive. This data is a valuable contribution to the literature.  It would be helpful to report the financial costs as well in the supporting information.

Authors: Thanks for the encouraging comment and the valuable suggestion to report financial costs. As we felt that they were an important addition, we created an additional panel in Table 5. We added explanatory text to the methods (around lines 302 and 344), results (around line 414 and Table 5) and conclusions (around line 611).

1.2	My major concern is with the regression analysis approach used to capture the effectiveness of the interventions – which needs to be further justified. For example, it is possible that the implementation period was not long enough to show the full long-term incremental benefits of vector control or that vector control should be targeted to transmission hotspots/areas with higher baseline endemicity (both potentially reducing the number of MDA rounds needed in the future). I believe this needs to be made clearer in the discussion.

Authors: Based on these thoughtful questions, we have acknowledged this limitation and the options for future targeting around lines 577-593.

1.3	In addition, several aspects of the methodology need to be made clearer
(such as the perspective and discount rate). I suggest the authors follow
the CHEERS checklist.

Authors: To clarify, we have added explanations of our perspective (around lines 338-352) and discounting (around lines 351-355). We have also included numerous other clarifications throughout the text. Although we did not include a CHEERS checklist, we have checked that our manuscript includes all the required elements.

1.4	A sensitivity analysis should be conducted.

Authors We have added this as Supporting Information S6.

1,5	Minor comments. In the methods, you say you annualizing capital costs – what discount rate was used.

[Authors: we used 3% as explained in comment 1.3. Please see around line 308.]

1.6	“With MDA offered in all arms, all reduced LF prevalence substantially and
significantly from 2010 to 2013”. A little unclear to have both "substantially and significantly"

[Authors: we deleted “significantly” around line 52]

1.7	Value per DALY should be per DALY averted instead - The ICER for MDA is
$112 per DALY should be The ICER for MDA is $112 per DALY averted.

[Authors: We edited the text accordingly around line 494.]

**Reviewer #2:**

2.1	General comments. The present manuscript deals with the results of a study, conducted as a follow-up of cost-effectiveness of Mass Drug Administration (MDA) with or without vector control (Krishnamoorthy et al., 2002) in 36 different villages in Kallakurichi health district between 2010 and 2013. While MDA was administered by the State Health department as per the national guidelines, vector control was carried out by the research team with community participation. Though the findings were similar to the earlier publication of cost-effectiveness of MDA and MDA+VC, the current publication has additionally used filarial antigenemia and estimated DALYs averted. MDA cost analysis tool (MDA-CAT) and VC cost analysis tool (VC-CAT) were employed, to enter the cost data and perform cost analyses. Two interventions against the vector, one with expanded polystyrene beads (EPBs) in breeding places and the other integrated this with pyrethroid impregnated curtains (PIC) were carried out to compare the outcome of MDA with vector control strategies. The study also confirmed that MDA is more cost-effective in achieving interruption of transmission. **The manuscript with added outcome indicators is recommended for publication.** Though the manuscript is published after a gap of 10 years **it provides valuable information on the impact of vector control interventions.** [Authors: Thank you.].

2.2	The present document added another arm with integrated vector control. Vector control included only treatment of cesspits with polystyrene beads (VCS) and integrated with use of insecticide treated curtains VCI). Earlier trial used application of Bacillus sphaericus in breeding places. It is understood that this intervention was not used in the **present study.** [Authors: we expanded the description of the present study’s intervention around lines 193-229]

Reviewer 2 specific comments:

1. Line 58 – add after additional benefit “on infection prevalence” [Authors: we added text]
2. Line 60 – refer to the latest reference on WHO-targeted NTDs for control. [Authors: latest reference now provided, see lines 84-87]
3. Line 61-63 – Indian states started MDA programme in 2004 and earlier studies were trails with MDA in selected districts. This can be modified. [Authors: we modified this and provided relevant references in lines 78-79]
4. Replace the reference 7 with the latest one “Vol. 98, No. 41, pp. 489 – 502” - 19 countries and territories (Cambodia, the Cook Islands, Egypt, Kiribati, Maldives, Malawi, Marshall Islands, Niue, Palau, Sri Lanka, Thailand, Togo, Tonga, Vanuatu, Viet Nam, Wallis and Futuna, Yemen, Lao PDR and Bangladesh) are now acknowledged as achieving elimination of lymphatic filariasis as a public health problem. [Authors: we updated and provided the new reference, now reference 9]
5. Line 79 – replace “elimination from 20 countries” with “19 countries validated for elimination of LF as a public health problem (Vol. 98, No. 41, pp. 489 – 502 and reference 8) [Authors: Thank you. We updated using the reference to the 2023 publication, now reference 9.]
6. Line 81 – add “and elimination of LF in 2004” after 1955. [Authors: we updated the text to add this phrase around line 89]
7. Line 81 – replace “Control” by elimination [Authors: we replaced text around line 89]
8. Line 86-89 – Rephrase the sentences as “Initially the MDA program administered a single dose of diethylcarbamazine (DEC) to each eligible person and subsequently administered DEC plus albendazole annually to the whole population of districts (implementation units) where LF was endemic [10, 11]”. [Authors: we rephrased around lines 94-96]
9. Line 118 - Add a suitable reference after COVID-19 [Authors: We added a WHO citation in lines 126-130.]
10. Line 128 – add “present” after “study” [We revised the text]
11. Line 220 – rephrase as “anti-filarial drugs with DEC and albendazole…” [Authors: We added this text around line 194]
12. Line 230 – add “unused” before wells [Authors: We added this text around line 205]
13. Line 238 – what modification was done in unused wells? [Authors: We described the modification to wells around lines 218-219]
14. Line 315 – the method of calculating DALY per 1000 population averted can be given [Authors: We describe the methods around lines 387-397]
15. Line 352 – add “infection” after LF [Authors: We added this]
16. Line 355 – justify use of LF prevalence data for calculating DALYs per person averted [Authors: We added the justification]
17. Figure 5 – correct the y axis title as AgP [Authors: We corrected this]
18. Line 416 – 418 & Figure 4 and 5 - what is the explanation for significant reduction in AgP following the intervention? [Authors: We answered this around lines 440-445]
19. Line 428 – the benefit of reduction in vector density following VCS and VCI can be mentioned [Authors: We explained this around lines 537-543 of Discussion Section]
20. Line 432 - provide the source of data for prevalence of infection in India [Authors: The source of data is the Institute of Health Metrics, 2023, reference 58]
21. Line 434 and Figure 6 – the limitation of using prevalence data from sample survey in selected sites every year can be explained, particularly for India [Authors: this is now written around Lines 545-550]
22. Line 436 – how to justify estimating people affected by LF based on prevalence which is expected to be done only from endemic areas [Authors: this explanation is written around lines 551-556]
23. Table 7 – how do we explain the change in AgP from 2010 (baseline) and 2013 (post intervention) [We note that the reduction in the study area is consistent with national trends around line 484, citing reference 4, and indicate around line 532 that Tamil Nadu’s effective delivery of preventive interventions probably deserves credit around line 532]
24. Line 445 – justify the extrapolation of data from 36 villages to the whole of nation

[Authors: Acknowledging this have endeavored to be more explicit about the study’s limitations, especially around lines 575-581.]

25. Line 455 – add “infection” after LF [Authors: we added this]
26. Line 458 – replace the word “affected” by infected [Authors: we replaced this]
27. Line 461 – add “Kalakurichi district” after the study [Authors: we added this]
28. Line 468 – add “selected” before endemic (MDA was conducted in selected districts in 1996-98 and LF elimination programme was introduced in India by 2004) [added]
29. Line 469 – replace 2001 by 2007 as nation-wide co-administration of DEC+albendazole was introduced in India by 2007. (trial with DEC+albendazole, as co-administration was done in 2001 in selected districts in Tamil Nadu) [Authors: We adjusted our text]
30. Line 472 – add “Kalakurich health unit district of” before Villupuram [Authors: we added Kallakurichi]
31. Line 476 – rephrase the sentence as “reached the target levels of AgP<2% in children by transmission assessment survey” [Authors: we rephrased this around line 513]
32. Line 491 – add replace “the only” with one of the comparative studies [Authors: we added the comparative study around lines 520-525]
33. Line 493 – replace “progress” by “impact” [Authors: we replaced this]
34. Line 495 – add “short term” before study [Authors: we added this]
35. Line 506 – add “reduced” before MfP and replace adequately controlled with “in selected sites” [Authors: we added and replaced text]
36. Line 507 – replace “most of the” with “at least 99” districts by giving reference “Roy N. Elimination of lymphatic filariasis – India: Updates and way forward. MJMS. 2018; 3(2): 1-3”. [Authors: we revised the text and added the new citation as reference 66]s
37. Line 508 – replace the sentence “However, 12 such villages in non-endemic areas had MfP between 3.2% and 11.2% with “Remapping of non-endemic areas in India [60] suggest prevalence of infection above 1% in 12 such sites” (consider deleting this paragraph line 505-509 which is not relevant here) [Authors: we replaced text and deleted the unneeded paragraph]
38. Line 511 – provide data on reduction in mosquito nuisance [Authors: we added data on reduction in biting and vector density around lines 557-561.]
39. Line 535 – add “in achieving the interruption of transmission” after MDA [Authors: we revised the text]

**Reviewer #3:**

3.1	General comments: The objectives of the study are clearly stated and the study design is appropriate. The study population is described well and appropriate, and the sample size is sufficient for testing the study hypothesis. Appropriate statisical analysis of the data was done to arrive at conclusions.

[Authors: Thank you.]

3.1	General comments. The manuscript deals with a study on the cost-effectiveness of different interventions for the elimination of lymphatic filariasis which is a major public health problem in many tropical countries. In view of the persisting Mf prevalence even after several rounds, due to sub-optimal coverage and compliance, vector control options including integrated methods are being explored as additional tools. Cost considerations become key issues when adapting multiple interventions and, in this context, the present study is very relevant. The study is well conducted and the cost analysis is done appropriately. I have some major and minor comments which need to be addressed before the manuscript is accepted, which are as below:

[Authors: we comment on each point below]

3.2	Major comments: The conclusion of the study is that the MDA was effective and very cost-effective for eliminating LF, leaving little scope for vector control for further improvement. This is in contrast to the statement in Authors summary which concludes that the study had limited ability to detect further improvements against the low level of LF endemicity in the study areas. It is expected that when the endemicity is low it will be difficult to discern differences between effectiveness of different control methods. This is a major issue of the study which has a bearing on the conclusion.

[Authors: Thanks for pointing out the contradiction. We rewrote the summary to address this major issue.]

3.3	Earlier studies have concluded that MDA augmented by VC reduced both the annual transmission potential and the transmission intensity index of LF to zero in Tamil Nadu villages with an added cost of US$0.85 per person annually [25]. But, the present study did not find any additional . This needs to be explained.

Authors: The unexpected limited power of the study was discussed around lines 121-124

3.4	Lines 499-509: This is another major lacuna. The important data on coverage and compliance of drugs administered, and coverage of breeding habitats for VC which has not been considered. In the absence this data comparing the effectiveness and cost-effectiveness of control tools may not be appropriate.

Authors: We agree that better adherence had previously been problematic. We acknowledge that but present data that the situation has improved in recent years. The success in our MDA villages confirms this. Please see approximate lines 593-615

3.4.	Lines 154-155: The statement should be modified as ‘The World Health Organization defined the eligibility of communities for MDA treatment as the prevalence of filarial parasites (MfP) or antigens of more than 1% and 2%, respectively’.

Authors: Thanks for the clarification. The text has been corrected accordingly around line 163

3.5.	Lines 156-158: What were the base line Mf prevalence and antigen
prevalence.

Authors: We added Supporting information S1 to describe the base year and final year’s MfP and AgP prevalence

3.6.	Line 181: The drug dosage regimens can be stated.

Authors: We added this description around lines 198-201

3.7.	Line 229: Why was the range 350–400 g/m² used?

Authors: When we checked, we found that this is the traditional approach of CRME practiced since mid-1990s. We added this clarification, citing Sunish et al 2007 (reference number 31) around line 239.

3.8.	Lines 298-299: Whether the two cost analysis tools: mass drug administration cost analysis tool (MDA-CAT) and VC cost analysis tool (VC-CAT), were validated prior to their use in the study?

Authors: Yes, the economists within the research team discussed with the India Government’s senior colleagues the analysis tools and conducted multiple rounds of validations and modifications (we added this clarification around line 280.)

3.9.	Lines 356-358: The interventions are targeted against filarial parasite and hence whether is it appropriate to calculate DALYs averted which takes into account the disabilities (lymphedema and hydrocele), that too when the interventions is just for 3 years.

Authors: We utilized the Institute for Health Metrics and Evaluation's (IHME) disease-specific DALY estimates (reference 58), which inherently account for associated disabilities (lymphedema and hydrocele). Although our intervention lasted three years, the villages achieved elimination targets. We project that once elimination is reached, the population will no longer experience the DALY losses previously associated with LF transmission.
