# Supplementary material for: Cost-effectiveness of vector control for supplementing mass drug administration for eliminating lymphatic filariasis in India
Source: PLoS Negl Trop Dis. 2024 Dec 4;18(12):e0011835. doi: 10.1371/journal.pntd.0011835 (PMC11649082; doi:10.1371/journal.pntd.0011835)
Supplement: Attachment — Submitted filename: Response04.docx [file pntd.0011835.s005.docx]

**Response to reviewers regarding**

**Cost-effectiveness of vector control for supplementing mass drug administration for eliminating lymphatic filariasis in India**

Note. For clarity, we have numbered the points and begin our responses with the label: “Authors.” Line numbers refer to the clean version

1. Line 83, It would make sense to introduce WHO’s Global Programme to eliminate LF (GPELF), before mentioning the national program and to explain the advisory role of WHO for LF elimination. GPELF was first mentioned in line 234.

Authors: Done (see line 83).

2. Line 201, C. quinquefasciatus.

Authors: Spelling and formatting updated (line 199).

3. Line 205, a brief introduction into the results (what was the question studied in this paragraph) after a long M& M section would be helpful instead of repeating Table 2 shows, Table 3 shows, Table 4 shows, Table 5 shows ….

Authors. We rewrote the subsection “Economic cost of interventions” as suggested (line 401).

4. Line 1984. The abstract (Line 44) explains the study arms MDA, VCS and VCI. But what is PIC? EBP was introduced in line 108, but not PIC. The heavy and inconsistent use of abbreviation makes the ms difficult to read.

Authors: To clarify the manuscript, we removed all rarely used abbreviations. We now introduce the two interventions around the same place in the manuscript (EPB at line 196 and PIC at line 213) and explain their relation to VCS and VCI. We also revised the discussion extensively, adding subheadings, and updating citations, and made other clarifying edits throughout the manuscript.

5. Line 314. Why is the explanation of VCS and VCI different from line 44?

Authors: We revised line 45 in the abstract so it is now consistent with the man text (lines 195 and 309).

6. Line 520. Are completed MDA rounds also effective MDA rounds with more that 65% coverage?

Authors: We added a sentence explaining the considerations indicating that the MDA rounds were effective (lines 613-617).

7. Lines 524. What were the results of the preTAS and the TAS-1? What were the results of TAS-2 and TAS-3?

Authors: Please see response to previous comment and lines 613-617.

8. Line 527. What is meant by ‘district’? HUD? What was the evaluation unit for TAS (HUD hat 3.5 million residents)?

Authors: On line 147, we defined the acronym HUD. After the study’s intervention period, Kallalkurichi District was created out of a portion of the former Viluppurum District. Accordingly, on line 150 we wrote “areas now forming….”

9. Line 565. 2023,, VCS….

Authors: We corrected the punctuation (line 561)

10. Line 628. India is already extensively using IDA triple drug MDA

Authors: We reported the introduction of triple drug therapy in some sites (line 625). We changed from “possibly” to “increasingly” so the phrase now reads “increasingly with triple-drug therapy….” (line 677).
